# Supplementary material for: Cartilage Regeneration Using Human Umbilical Cord Blood Derived Mesenchymal Stem Cells: A Systematic Review and Meta-Analysis
Source: Medicina (Kaunas). 2022 Dec 6;58(12):1801. doi: 10.3390/medicina58121801 (PMC9786930; doi:10.3390/medicina58121801)
Supplement: Supplementary file 1 [file medicina-58-01801-s001.zip › Supplementary file S1.pdf]

Supplementary file S1. Keywords and search result

Database	Search	Keywords	Results
**PubMed**	#1	"Cartilage, Articular"[Mesh]	32,176
	#2	"Cartilage, Articular"[TW] OR "Articular Cartilage"[TW] OR "Articular Cartilages"[TW] OR "Cartilages, Articular"[TW] OR "knee cartilage defect"[TW] OR "cartilage, joint"[TW] OR "joint cartilage"[TW]	41,665
	#3	"Knee Joint"[Mesh]	68,467
	#4	"Knee Joint"[TW] OR "Joint, Knee"[TW] OR "Joints, Knee"[TW] OR "Knee Joints"[TW] OR "Superior Tibiofibular Joint"[TW] OR "Joint, Superior Tibiofibular"[TW] OR "Joints, Superior Tibiofibular"[TW] OR "Superior Tibiofibular Joints"[TW] OR "Tibiofibular Joint, Superior"[TW] OR "Tibiofibular Joints, Superior"[TW]	76,539
	#5	"Osteoarthritis, Knee"[Mesh]	24,984
	#6	"Osteoarthritis, Knee"[TW] OR "Knee Osteoarthritides"[TW] OR "Knee Osteoarthritis"[TW] OR "Osteoarthritis of Knee"[TW] OR "Osteoarthritis of the Knee"[TW] OR "osteoarthritic knee"[TW] OR "osteoarthritic knees"[TW] OR "arthrosis, knee"[TW] OR "femorotibial arthrosis"[TW] OR "gonarthrosis"[TW] OR "knee arthrosis"[TW] OR "knee joint arthrosis"[TW] OR "knee joint osteoarthritis"[TW] OR "knee osteo-arthritis"[TW] OR "knee osteo-arthrosis"[TW] OR "knee osteoarthrosis"[TW] OR "osteoarthrosis, knee"[TW]	31,576
	**#7**	**#1 OR #2 OR #3 OR #4 OR #5 OR #6**	**130,707**
	#8	"human umbilical cord blood-derived mesenchymal stem cell"[TW] OR "human umbilical cord blood-derived mesenchymal stem cells"[TW] OR "hUCB-MSC"[TW] OR "hUCB-MSCs"[TW] OR "umbilical cord blood-derived mesenchymal stem cell"[TW] OR "umbilical cord blood-derived mesenchymal stem cells "[TW] OR "UCB-MSC"[TW] OR "UCB-MSCs"[TW] OR "umbilical cord blood stem cell"[TW] OR "umbilical cord blood stem cells"[TW] OR "UC-mesenchymal stem cells"[TW] OR "UC-MSC"[TW] OR "umbilical cord mesenchymal stem cell"[TW] OR "umbilical cord (UC) -mesenchymal stem cells (MSC)"[TW] OR "umbilical cord mesenchymal stem cells"[TW] OR "umbilical cord MSC"[TW] OR "umbilical cord MSC (UC-MSC)"[TW] OR	2,752

		"umbilical cord-derived mesenchymal stem cell"[TW] OR "umbilical cord-derived mesenchymal stem cells"[TW]	
	#9	"UC-MSC transplantation"[TW] OR "umbilical cord-derived mesenchymal stem cell transplantation"[TW] OR "umbilical cord (UC) mesenchymal stem cells transplantation (MSCT)"[TW] OR "umbilical cord (UC) -derived mesenchymal stem cells (MSCs)"[TW] OR "UCMSCT"[TW] OR "UC-MSCT"[TW]	53
	#10	"Cord Blood Stem Cell Transplantation"[Mesh]	3,602
	#11	"Cord Blood Stem Cell Transplantation"[TW] OR "Stem Cell Transplantation, Placental Blood"[TW] OR "Umbilical Cord Blood Stem Cell Transplantation"[TW] OR "Placental Blood Stem Cell Transplantation"[TW] OR "Stem Cell Transplantation, Cord Blood"[TW] OR "Blood Stem Cell Transplantation, Umbilical Cord"[TW] OR "cord blood transplantation"[TW] OR "umbilical cord stem cell transplantation"[TW]	4,592
	**#12**	**#8 OR #9 OR #10 OR #11**	**7,044**
	**#13**	**#7 AND #12**	**62**
**Database**	**Search**	**Keywords**	**Results**
**EMBASE**	#1	"articular cartilage"/exp	32,740
	#2	"Cartilage, Articular":ti,ab,kw,de OR "Articular Cartilage":ti,ab,kw,de OR "Articular Cartilages":ti,ab,kw,de OR "Cartilages, Articular":ti,ab,kw,de OR "knee cartilage defect":ti,ab,kw,de OR "cartilage, joint":ti,ab,kw,de OR "joint cartilage":ti,ab,kw,de	43,553
	#3	"knee"/exp	77,583

	#4	"Knee Joint":ti,ab,kw,de OR "Joint, Knee":ti,ab,kw,de OR "Joints, Knee":ti,ab,kw,de OR "Knee Joints":ti,ab,kw,de OR "Superior Tibiofibular Joint":ti,ab,kw,de OR "Joint, Superior Tibiofibular":ti,ab,kw,de OR "Joints, Superior Tibiofibular":ti,ab,kw,de OR "Superior Tibiofibular Joints":ti,ab,kw,de OR "Tibiofibular Joint, Superior":ti,ab,kw,de OR "Tibiofibular Joints, Superior":ti,ab,kw,de	38,747
	#5	"knee osteoarthritis"/exp	41,073
	#6	"Osteoarthritis, Knee":ti,ab,kw,de OR "Knee Osteoarthritides":ti,ab,kw,de OR "Knee Osteoarthritis":ti,ab,kw,de OR "Osteoarthritis of Knee":ti,ab,kw,de OR "Osteoarthritis of the Knee":ti,ab,kw,de OR "osteoarthritic knee":ti,ab,kw,de OR "osteoarthritic knees":ti,ab,kw,de OR "arthrosis, knee":ti,ab,kw,de OR "femorotibial arthrosis":ti,ab,kw,de OR "gonarthrosis":ti,ab,kw,de OR "knee arthrosis":ti,ab,kw,de OR "knee joint arthrosis":ti,ab,kw,de OR "knee joint osteoarthritis":ti,ab,kw,de OR "knee osteo-arthritis":ti,ab,kw,de OR "knee osteo-arthrosis":ti,ab,kw,de OR "knee osteoarthrosis":ti,ab,kw,de OR "osteoarthrosis, knee":ti,ab,kw,de	45,268
	**#7**	**#1 OR #2 OR #3 OR #4 OR #5 OR #6**	**164,232**
	#8	"human umbilical cord blood-derived mesenchymal stem cell":ti,ab,kw,de OR "human umbilical cord blood-derived mesenchymal stem cells":ti,ab,kw,de OR "hUCB-MSC":ti,ab,kw,de OR "hUCB-MSCs":ti,ab,kw,de OR "umbilical cord blood-derived mesenchymal stem cell":ti,ab,kw,de OR "umbilical cord blood-derived mesenchymal stem cells ":ti,ab,kw,de OR "UCB-MSC":ti,ab,kw,de OR "UCB-MSCs":ti,ab,kw,de OR "umbilical cord blood stem cell":ti,ab,kw,de OR "umbilical cord blood stem cells":ti,ab,kw,de OR "UC-mesenchymal stem cells":ti,ab,kw,de OR "UC-MSC":ti,ab,kw,de OR "umbilical cord mesenchymal stem cell":ti,ab,kw,de OR "umbilical cord (UC) -mesenchymal stem cells (MSC)":ti,ab,kw,de OR "umbilical cord mesenchymal stem cells":ti,ab,kw,de OR "umbilical cord MSC":ti,ab,kw,de OR "umbilical cord MSC (UC-MSC)":ti,ab,kw,de OR "umbilical cord-derived	4,374

		mesenchymal stem cell":ti,ab,kw,de OR "umbilical cord-derived mesenchymal stem cells":ti,ab,kw,de	
	#9	"UC-MSC transplantation":ti,ab,kw,de OR "umbilical cord-derived mesenchymal stem cell transplantation":ti,ab,kw,de OR "umbilical cord (UC) mesenchymal stem cells transplantation (MSCT)":ti,ab,kw,de OR "umbilical cord (UC) -derived mesenchymal stem cells (MSCs)":ti,ab,kw,de OR "UCMSCT":ti,ab,kw,de OR "UC-MSCT":ti,ab,kw,de	91
	#10	"cord blood stem cell transplantation"/exp	6,901
	#11	"Cord Blood Stem Cell Transplantation":ti,ab,kw,de OR "Stem Cell Transplantation, Placental Blood":ti,ab,kw,de OR "Umbilical Cord Blood Stem Cell Transplantation":ti,ab,kw,de OR "Placental Blood Stem Cell Transplantation":ti,ab,kw,de OR "Stem Cell Transplantation, Cord Blood":ti,ab,kw,de OR "Blood Stem Cell Transplantation, Umbilical Cord":ti,ab,kw,de OR "cord blood transplantation":ti,ab,kw,de OR "umbilical cord stem cell transplantation":ti,ab,kw,de	7,638
	**#12**	**#8 OR #9 OR #10 OR #11**	**11,382**
	**#13**	**#7 AND #12**	**120**

Database	Search	Keywords	Results
**Cochrane Library**	#1	[mh "Cartilage, Articular"]	306
	#2	"Cartilage, Articular":ti,ab,kw OR "Articular Cartilage":ti,ab,kw OR "Articular Cartilages":ti,ab,kw OR "Cartilages, Articular":ti,ab,kw OR "knee cartilage defect":ti,ab,kw OR "cartilage, joint":ti,ab,kw OR "joint cartilage":ti,ab,kw	1,000
	#3	[mh "Knee Joint"]	3,664

	#4	"Knee Joint":ti,ab,kw OR "Joint, Knee":ti,ab,kw OR "Joints, Knee":ti,ab,kw OR "Knee Joints":ti,ab,kw OR "Superior Tibiofibular Joint":ti,ab,kw OR "Joint, Superior Tibiofibular":ti,ab,kw OR "Joints, Superior Tibiofibular":ti,ab,kw OR "Superior Tibiofibular Joints":ti,ab,kw OR "Tibiofibular Joint, Superior":ti,ab,kw OR "Tibiofibular Joints, Superior":ti,ab,kw	6,734
	#5	[mh "Osteoarthritis, Knee"]	5,094
	#6	"Osteoarthritis, Knee":ti,ab,kw OR "Knee Osteoarthritides":ti,ab,kw OR "Knee Osteoarthritis":ti,ab,kw OR "Osteoarthritis of Knee":ti,ab,kw OR "Osteoarthritis of the Knee":ti,ab,kw OR "osteoarthritic knee":ti,ab,kw OR "osteoarthritic knees":ti,ab,kw OR "arthrosis, knee":ti,ab,kw OR "femorotibial arthrosis":ti,ab,kw OR "gonarthrosis":ti,ab,kw OR "knee arthrosis":ti,ab,kw OR "knee joint arthrosis":ti,ab,kw OR "knee joint osteoarthritis":ti,ab,kw OR "knee osteo-arthritis":ti,ab,kw OR "knee osteo-arthrosis":ti,ab,kw OR "knee osteoarthrosis":ti,ab,kw OR "osteoarthrosis, knee":ti,ab,kw	11,705
	**#7**	**#1 OR #2 OR #3 OR #4 OR #5 OR #6**	**16,258**
	#8	"human umbilical cord blood-derived mesenchymal stem cell":ti,ab,kw OR "human umbilical cord blood-derived mesenchymal stem cells":ti,ab,kw OR "hUCB-MSC":ti,ab,kw OR "hUCB-MSCs":ti,ab,kw OR "umbilical cord blood-derived mesenchymal stem cell":ti,ab,kw OR "umbilical cord blood-derived mesenchymal stem cells ":ti,ab,kw OR "UCB-MSC":ti,ab,kw OR "UCB-MSCs":ti,ab,kw OR "umbilical cord blood stem cell":ti,ab,kw OR "umbilical cord blood stem cells":ti,ab,kw OR "UC-mesenchymal stem cells":ti,ab,kw OR "UC-MSC":ti,ab,kw OR "umbilical cord mesenchymal stem cell":ti,ab,kw OR "umbilical cord (UC) -mesenchymal stem cells (MSC)":ti,ab,kw OR "umbilical cord mesenchymal stem cells":ti,ab,kw OR "umbilical cord MSC":ti,ab,kw OR "umbilical cord MSC (UC-MSC)":ti,ab,kw OR "umbilical cord-derived mesenchymal stem cell":ti,ab,kw OR "umbilical cord-derived mesenchymal stem cells":ti,ab,kw	277

	#9	"UC-MSC transplantation":ti,ab,kw OR "umbilical cord-derived mesenchymal stem cell transplantation":ti,ab,kw OR "umbilical cord (UC) mesenchymal stem cells transplantation (MSCT)":ti,ab,kw OR "umbilical cord (UC) -derived mesenchymal stem cells (MSCs)":ti,ab,kw OR "UCMSCT":ti,ab,kw OR "UC-MSCT":ti,ab,kw	14
	#10	[mh "Cord Blood Stem Cell Transplantation"]	27
	#11	"Cord Blood Stem Cell Transplantation":ti,ab,kw OR "Stem Cell Transplantation, Placental Blood":ti,ab,kw OR "Umbilical Cord Blood Stem Cell Transplantation":ti,ab,kw OR "Placental Blood Stem Cell Transplantation":ti,ab,kw OR "Stem Cell Transplantation, Cord Blood":ti,ab,kw OR "Blood Stem Cell Transplantation, Umbilical Cord":ti,ab,kw OR "cord blood transplantation":ti,ab,kw OR "umbilical cord stem cell transplantation":ti,ab,kw	150
	**#12**	**#8 OR #9 OR #10 OR #11**	**411**
	**#13**	**#7 AND #12**	**20**

Database	Search	Keywords	Results
**Web of Science**	#1	TS=("Cartilage, Articular" OR "Articular Cartilage" OR "Articular Cartilages" OR "Cartilages, Articular" OR "knee cartilage defect" OR "cartilage, joint" OR "joint cartilage")	37,687
	#2	TS=("Knee Joint" OR "Joint, Knee" OR "Joints, Knee" OR "Knee Joints" OR "Superior Tibiofibular Joint" OR "Joint, Superior Tibiofibular" OR "Joints, Superior Tibiofibular" OR "Superior Tibiofibular Joints" OR "Tibiofibular Joint, Superior" OR "Tibiofibular Joints, Superior")	27,421
	#3	TS=("Osteoarthritis, Knee" OR "Knee Osteoarthritides" OR "Knee Osteoarthritis" OR "Osteoarthritis of Knee" OR "Osteoarthritis of the Knee" OR "osteoarthritic knee" OR "osteoarthritic knees" OR "arthrosis, knee" OR "femorotibial arthrosis" OR "gonarthrosis" OR "knee arthrosis" OR "knee joint arthrosis" OR "knee joint osteoarthritis" OR "knee osteo-arthritis" OR "knee osteo-arthrosis" OR "knee osteoarthrosis" OR "osteoarthrosis, knee")	30,180
	**#4**	**#1 OR #2 OR #3**	**84,571**

	#5	TS=("human umbilical cord blood-derived mesenchymal stem cell" OR "human umbilical cord blood-derived mesenchymal stem cells" OR "hUCB-MSC" OR "hUCB-MSCs" OR "umbilical cord blood-derived mesenchymal stem cell" OR "umbilical cord blood-derived mesenchymal stem cells " OR "UCB-MSC" OR "UCB-MSCs" OR "umbilical cord blood stem cell" OR "umbilical cord blood stem cells" OR "UC-mesenchymal stem cells" OR "UC-MSC" OR "umbilical cord mesenchymal stem cell" OR "umbilical cord (UC) - mesenchymal stem cells (MSC)" OR "umbilical cord mesenchymal stem cells" OR "umbilical cord MSC" OR "umbilical cord MSC (UC-MSC)" OR "umbilical cord-derived mesenchymal stem cell" OR "umbilical cord-derived mesenchymal stem cells")	3,027
	#6	TS=("UC-MSC transplantation" OR "umbilical cord-derived mesenchymal stem cell transplantation" OR "umbilical cord (UC) mesenchymal stem cells transplantation (MSCT)" OR "umbilical cord (UC) -derived mesenchymal stem cells (MSCs)" OR "UCMSCT" OR "UC-MSCT")	53
	#7	TS=("Cord Blood Stem Cell Transplantation" OR "Stem Cell Transplantation, Placental Blood" OR "Umbilical Cord Blood Stem Cell Transplantation" OR "Placental Blood Stem Cell Transplantation" OR "Stem Cell Transplantation, Cord Blood" OR "Blood Stem Cell Transplantation, Umbilical Cord" OR "cord blood transplantation" OR "umbilical cord stem cell transplantation")	4,639
	**#8**	**#5 OR #6 OR #7**	**7,575**
	**#9**	**#7 AND #12**	**76**
**Database**	**Search**	**Keywords**	**Results**
**Scopus**	#1	TITLE-ABS-KEY("Cartilage, Articular" OR "Articular Cartilage" OR "Articular Cartilages" OR "Cartilages, Articular" OR "knee cartilage defect" OR "cartilage, joint" OR "joint cartilage")	50,945
	#2	TITLE-ABS-KEY("Knee Joint" OR "Joint, Knee" OR "Joints, Knee" OR "Knee Joints" OR "Superior Tibiofibular Joint" OR "Joint, Superior Tibiofibular" OR "Joints, Superior Tibiofibular" OR "Superior Tibiofibular Joints" OR "Tibiofibular Joint, Superior" OR "Tibiofibular Joints,	82,622

		Superior")	
	#3	TITLE-ABS-KEY("Osteoarthritis, Knee" OR "Knee Osteoarthritides" OR "Knee Osteoarthritis" OR "Osteoarthritis of Knee" OR "Osteoarthritis of the Knee" OR "osteoarthritic knee" OR "osteoarthritic knees" OR "arthrosis, knee" OR "femorotibial arthrosis" OR "gonarthrosis" OR "knee arthrosis" OR "knee joint arthrosis" OR "knee joint osteoarthritis" OR "knee osteo-arthritis" OR "knee osteo-arthrosis" OR "knee osteoarthrosis" OR "osteoarthrosis, knee")	44,610
	**#4**	**#1 OR #2 OR #3**	**150,106**
	#5	TITLE-ABS-KEY("human umbilical cord blood-derived mesenchymal stem cell" OR "human umbilical cord blood-derived mesenchymal stem cells" OR "hUCB-MSC" OR "hUCB-MSCs" OR "umbilical cord blood-derived mesenchymal stem cell" OR "umbilical cord blood-derived mesenchymal stem cells " OR "UCB-MSC" OR "UCB-MSCs" OR "umbilical cord blood stem cell" OR "umbilical cord blood stem cells" OR "UC-mesenchymal stem cells" OR "UC-MSC" OR "umbilical cord mesenchymal stem cell" OR "umbilical cord (UC) -mesenchymal stem cells (MSC)" OR "umbilical cord mesenchymal stem cells" OR "umbilical cord MSC" OR "umbilical cord MSC (UC-MSC)" OR "umbilical cord-derived mesenchymal stem cell" OR "umbilical cord-derived mesenchymal stem cells")	3,974
	#6	TITLE-ABS-KEY("UC-MSC transplantation" OR "umbilical cord-derived mesenchymal stem cell transplantation" OR "umbilical cord (UC) mesenchymal stem cells transplantation (MSCT)" OR "umbilical cord (UC) -derived mesenchymal stem cells (MSCs)" OR "UCMSCT" OR "UC-MSCT")	83
	#7	TITLE-ABS-KEY("Cord Blood Stem Cell Transplantation" OR "Stem Cell Transplantation, Placental Blood" OR "Umbilical Cord Blood Stem Cell Transplantation" OR "Placental Blood Stem Cell Transplantation" OR "Stem Cell Transplantation, Cord Blood" OR "Blood Stem Cell Transplantation, Umbilical Cord" OR "cord blood transplantation" OR "umbilical cord stem cell transplantation")	7,068

	**#8**	**#5 OR #6 OR #7**	**10,652**
	**#9**	**#7 AND #12**	**102**

**Database**	**Results**
PubMed	62
EMBASE	120
Cochrane Library	20
Web of Science	76
Scopus	102
Total	380
duplication	211
Final	169
