# Supplementary material for: Cartilage Regeneration Using Human Umbilical Cord Blood Derived Mesenchymal Stem Cells: A Systematic Review and Meta-Analysis
Source: Medicina (Kaunas). 2022 Dec 6;58(12):1801. doi: 10.3390/medicina58121801 (PMC9786930; doi:10.3390/medicina58121801)
Supplement: Supplementary file 1 [file medicina-58-01801-s001.zip › Supplementary file S2.pdf]

Supplementary file S2. Funnel plot of IKDC(a), WOMAC(b), VAS(c)

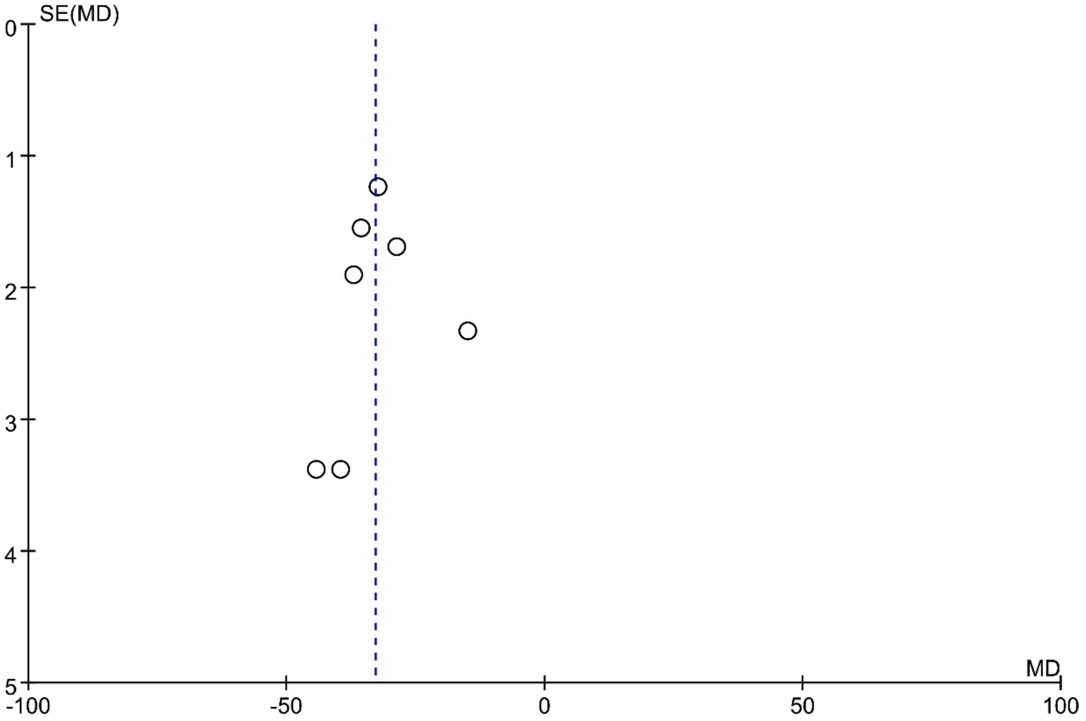

(a) Funnel plot of IKDC subjective score between preoperative IKDC and IKDC at final follow up.

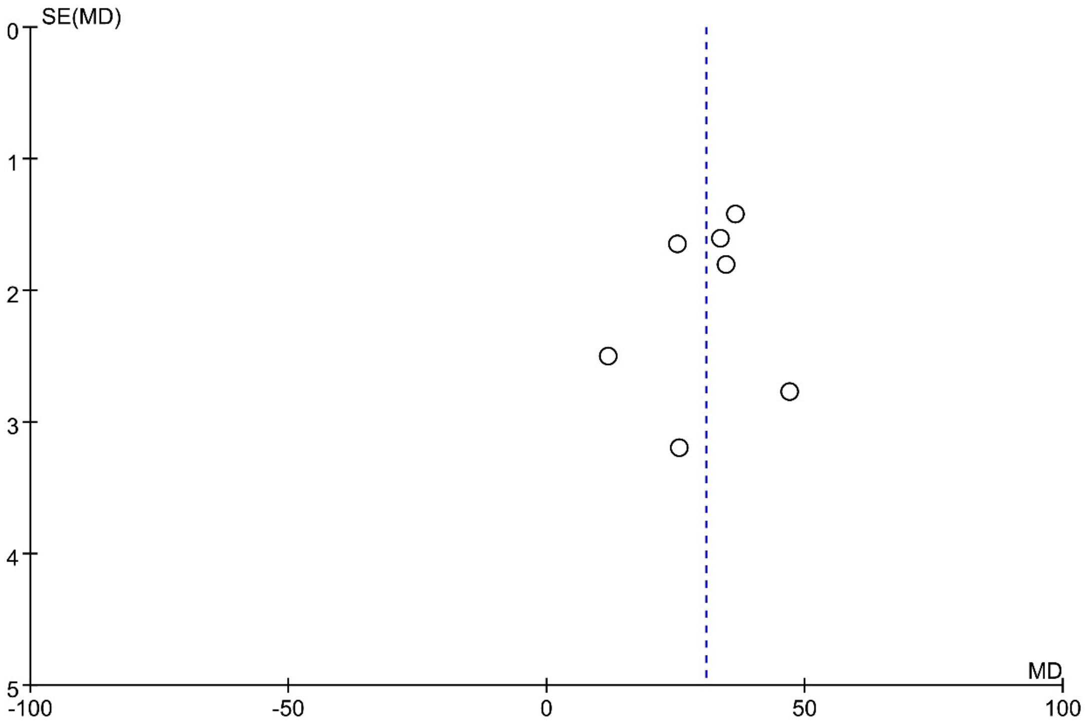

(b) Funnel plot of WOMAC between preoperative WOMAC and WOMAC at final follow up.

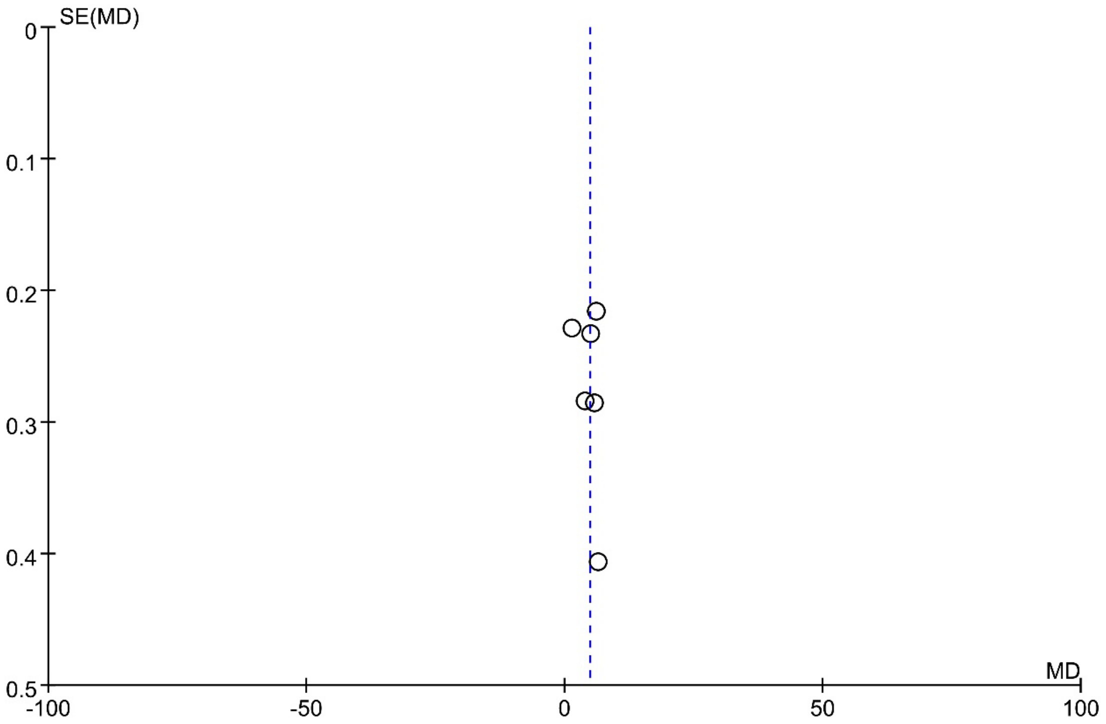

(c) Funnel plot of VAS between preoperative VAS and VAS at final follow up.
