# Supplementary material for: Cartilage Regeneration Using Human Umbilical Cord Blood Derived Mesenchymal Stem Cells: A Systematic Review and Meta-Analysis
Source: Medicina (Kaunas). 2022 Dec 6;58(12):1801. doi: 10.3390/medicina58121801 (PMC9786930; doi:10.3390/medicina58121801)
Supplement: Supplementary file 1 [file medicina-58-01801-s001.zip › Supplementary file S3.pdf]

Supplementary file S3. M-MOCART score & 2nd look arthroscopic findings of included studies.

study	follow up	Treatment	number of patients: MRI	M-MOCART		number of patients: 2nd look arthroscopy	ICRS grade at 2nd look arthroscopy				
							subgroup	1	2	3	4
chung 2021	mean 1.7Y (1.0-3.5Y)	hUCB-MSC		N/A		49	N/A	4	34	11	0
song(1) 2020	36.1 ± 6.4M (25-47M)			3-6M(mean3.8)	After 12M(mean21.2)		N/A				
		hUCB-MSC	34	30.58	55.44						
song(2) 2020	3Y	hUCB-MSC		N/A		125	N/A	73	37	15	0
song(3) 2020	26.7±1.8M (24–31M)	hUCB-MSC		N/A		14	N/A	6	8	0	0
Lee 2021	20.7 ± 6.1M	BMAC		N/A		42	MFC	1	18	12	11
							MTC	1	16	9	16
	15.6 ± 2.8M	hUCB-MSC		N/A		32	MFC	6	20	6	0
							MTC	2	24	5	1
Ryu 2022	2Y							ICRS repair grade score			
				1Y	2Y			1Y			
		BMAC	25	65.4 ± 13.46	70.20 ± 13.58	12	N/A	9.42 ± 1.83			
		hUCB-MSC	27	69.63 ± 13.37	73.7 ± 13.2	16	N/A	9.75 ± 2.05			

M-MOCART = Modified Magnetic Resonance Observation of Cartilage Repair Tissue, ICRS = International Cartilage Repair Society, Y = year, M = month, hUCB-MSC = human umbilical cord blood-derived mesenchymal stem cell, BMAC = bone marrow aspiration concentrate, MFC = medial femoral condyle, MTC = medial tibial condyle
